# Clinical and Biomechanical Determinants of Fixation Failure in Fifth Metatarsal Fractures: Implications for Surgical Decision-Making

**DOI:** 10.3390/jcm15051680

**Published:** 2026-02-24

**Authors:** Robert Daniel Dobrotă, Mark Pogărășteanu, Adrian Gheorghe Barbilian, Marius Moga

**Affiliations:** 1 Faculty of Medicine, Carol Davila University of Medicine and Pharmacy, 37 Dionisie Lupu Street, 020021 Bucharest, Romania; robert-daniel.dobrota@drd.umfcd.ro (R.D.D.); adrian.barbilian@umfcd.ro (A.G.B.); marius.moga@umfcd.ro (M.M.); 2 Dr. Carol Davila Central University Emergency Military Hospital, 010825 Bucharest, Romania

**Keywords:** fifth metatarsal fracture, Jones fracture, stress fracture, fixation failure, intramedullary screw, plate fixation, 3D printing, digital image correlation

## Abstract

**Objectives**: To provide a mechanism-oriented integration of clinical and biomechanical evidence regarding fixation failure in fifth metatarsal fractures, with particular emphasis on Jones and diaphyseal stress fractures, and to clarify the mechanical determinants that influence construct performance under physiologic gait-related loading. **Methods**: A narrative, concept-driven review was conducted focusing on experimental biomechanical investigations and clinically relevant outcome studies addressing cyclic shear, bending, torsion, interfragmentary gap behavior, and loading direction. Special attention was given to studies employing advanced experimental models, including three-dimensional printed anatomical constructs combined with digital image correlation (DIC), to evaluate fixation strategies under simulated gait-phase loading conditions. Literature selection was guided by thematic relevance to construct mechanics and clinical fixation outcomes rather than systematic retrieval criteria. **Results**: Available evidence indicates that fixation constructs relying predominantly on interfragmentary compression demonstrate increased sensitivity to imperfect reduction, interfragmentary gaps, and multidirectional cyclic shear forces, particularly during midstance loading. Experimental models suggest that loading angle and gap size significantly influence stress concentration and failure patterns. Plate-based and hybrid constructs may provide improved resistance to cyclic bending and shear in specific experimental conditions, maintain stability in the presence of small fracture gaps, and distribute mechanical loads more uniformly across the fracture site. These biomechanical characteristics may help explain reported clinical patterns of delayed union, refracture, and hardware failure in high-demand patients or in cases with cortical compromise. **Conclusions**: Fixation failure in fifth metatarsal fractures appears to result from the interaction between fracture morphology, patient-specific loading demands, and construct biomechanics. Mechanism-based integration of biomechanical findings with clinical context may support individualized surgical decision-making. However, given the heterogeneity of available clinical data and the inherent limitations of experimental models, biomechanical insights should be interpreted as hypothesis-generating and complementary to clinical judgment rather than prescriptive guidance.

## 1. Introduction

Metatarsal fractures account for approximately 6% of all fractures, with the fifth metatarsal being the most frequently involved. The most common categories affected by these types of fractures are athletes and especially young individuals engaged in sports that involve rapid directional changes, repetitive jumping and high-impact loading [1,2,3,4,5,6]. Beyond athletic populations, these fractures also occur in individuals with altered foot biomechanics, increased body mass or elderly people with osteoporosis. Because the lateral column of the foot is highly demanded during walking and running, even small increases in training intensity can predispose susceptible individuals to injury [7,8].

Despite high reported union rates with intramedullary screw fixation, particularly in elite athletes, there remains ongoing debate regarding optimal implant selection in cases characterized by cortical widening, stress-related bone changes, or high cyclic loading demands. While screw fixation is widely accepted and often considered the reference standard for acute Jones fractures, concerns persist regarding refracture and hardware failure in mechanically compromised environments. This uncertainty highlights the need for a clearer biomechanical framework to inform surgical decision-making. Recent motion-analysis studies have further demonstrated alterations in foot biomechanics before and after fifth metatarsal fracture during the running stance phase, reinforcing the dynamic mechanical environment involved in these injuries [9].

Because of its anatomical position and weight-bearing function, the fifth metatarsal is susceptible to three distinct fracture patterns: avulsion fractures, Jones fractures, and stress fractures. Each fracture type is characterized by specific biomechanical loading conditions, healing potential, and therapeutic implications.

**Figure 1:**
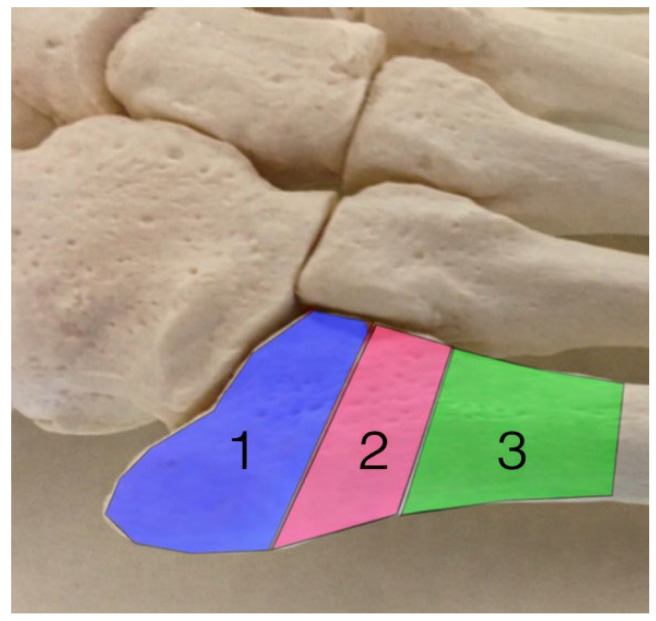
Types of fifth metatarsal fractures depending on the area they are located: 1—avulsion fractures; 2—jones fractures; 3—stress fractures.

These fractures are categorized in three biomechanically distinct zones (Figure 1), each characterized by specific loading patterns, vascular supply, and failure mechanisms, which directly influence fixation performance and healing potential:

- *Zone 1—Avulsion fractures*, caused by tensile forces from the peroneus brevis and lateral plantar fascia.
- *Zone 2—Jones fractures*, located at the metaphyseal–diaphyseal region.
- *Stress fractures* (*Zone 2–3*), resulting from repetitive microtrauma and fatigue failure [10,11,12,13].

The physical and vascular properties of each anatomic zone are important to understand how failure occurs in certain fixation devices subjected to physiological cyclic shear and how Zone II–III stress fractures can be different than acute fractures.

Additionally, the zoning system illustrated by Figure 1 provides the biomechanical basis for this review. The physical forces (tensile, bending, shear) experienced at each zone will vary with gait and explain why fixation devices will fail in one zone but perform well in others subjected to physiological cyclic loading.

The anatomy and blood supply of the proximal fifth metatarsal are crucial in deciding management based on fracture location. The uniqueness of these fractures also stems from how they develop. Insertion of the peroneal tendons at the tuberosity predisposes Zone I to avulsion under sudden inversion loads. Zone II’s limited vascular supply contributes to delayed union and non-union, while the progressively narrowing cortex toward Zone III is subjected to increasing bending and torsional stresses, promoting stress fracture development [14,15].

Stress fractures are particularly challenging because of high bending forces, repeated inversion stress, reduced blood flow and prolonged healing. Their particularity also lies in their mode of development. These result from fatigue accumulation, analogous to material fatigue: repeated loading cycles develop microfractures that propagate in the bone until its structure collapses. This process is accelerated in athletes, especially during midstance, when lateral bending forces peak [16,17].

**Figure 2:**
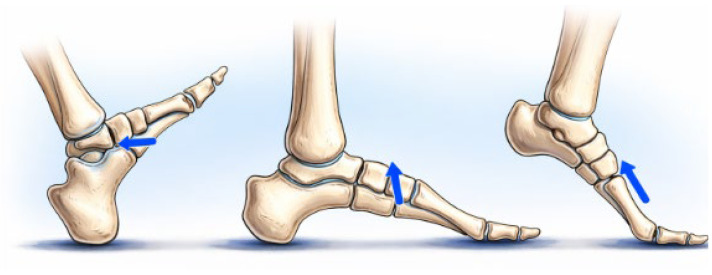
Resultant forces (arrow blue) applied to the fifth metatarsal during different phases of gait (original schematic based on published biomechanical data).

According to Dobrotă et al. [16], biomechanical analysis of gait has demonstrated that the fifth metatarsal is subjected to markedly different resultant forces during distinct phases of walking. Using the zonal biomechanical model illustrated in Figure 1 for the determination of resultant forces and their respective orientations, a resultant force of 85.15 N was found at heel strike and directed at an angle of 7° relative to the bone axis. During the intermediate support phase, a resultant force of 452.21 N was found and directed at an angle of 83° to the bone axis. A resultant force of 122.40 N was found during the propulsion phase and directed at an approximate angle of 150° to the bone axis. In order to perform a statistical analysis, the orientation of each resultant force was converted into its nearest principal direction (0°, 90° or 180°) as described in [16], thus allowing factorial analysis to be performed on this data set. Using the zonal biomechanical model illustrated in Figure 1 for the determination of resultant forces and their respective orientations, the resultant force vectors acting on the fifth metatarsal during the gait cycle are summarized in Figure 2.

Traditional cadaveric testing is limited by the variability of bones and the difficulty in reproducing physiologic shear and torsion. In contrast, 3D-printed bone models analyzed with digital image correlation (DIC) during gait phases produce clearer, more clinically useful outcomes. DIC provides detailed visualization of tensions around screw and plate connection points, plate hooks, and bone regions, being able to demonstrate the success of fixation during gait cycles.

These technologies allow for a better representation of how the mechanical environment in which the fifth metatarsal fractures develop and how the osteosynthesis methods perform mechanically during the gait cycle [2,3,10].

With this experimental combination, we are able to reproduce a fracture geometry and load it multidirectionally over the entire gait cycle to measure strains throughout the entire fracture region using digital image correlation. Therefore, this will allow us to perform a more standardized study of the interaction between implants and bone than what would be possible with cadavers. In recent years, three-dimensional modeling and experimental simulation have been developed and proposed as an additional tool to traditional cadaveric testing, especially when the goal is to have a controlled fracture geometry and to replicate loading conditions repeatedly. There have been previous studies in the area of orthopedic biomechanics that have suggested that such models could improve visualization of strain patterns and interactions between implants and bone. However, their clinical relevance is still being studied and interpreted cautiously [16,18,19].

Clinically, fixation failure of fifth metatarsal fractures remains a frequent source of reoperation, delayed return to sport, and patient dissatisfaction. Surgeons are often faced with the dilemma of choosing between intramedullary screws and plate-based constructs without clear guidance on how patient-related factors—such as activity level, foot alignment, bone quality, or early weight-bearing—interact with implant mechanics. Addressing this gap requires a clinically grounded understanding of how fixation constructs behave under physiologic loading conditions.

The main purpose of this review is to provide a biomechanical integration of the current state of managing fifth metatarsal fractures, specifically stress and Jones-type fractures. Although numerous clinical studies have reported on nonunion rates and complications of treating fifth metatarsal fractures, there is very little biomechanical explanation regarding why some fixation constructs fail under physiological loading conditions.

Specifically, this review aims to answer the following questions:

▪ What mechanical factors dominate failure and refracture risk in fifth metatarsal fractures?
▪ How do screws, plates, and hybrid constructs differ in their ability to withstand cyclic shear and bending?
▪ In what ways can advanced experimental models, such as 3D-printed bones combined with digital image correlation, improve the evaluation of implants beyond traditional cadaveric testing?

The review attempts to bridge the gap between biomechanical evidence and clinical decision-making. Therefore, the work presented here is intended to be a narrative biomechanical review, as opposed to a systematic review. The review presents a synthesis of mechanical principles, experimental results, and clinical implications relative to the fixation of fifth metatarsal fractures.

This review does not intend to provide a descriptive classification of fracture types or a training-oriented overview of 3-D printing.

## 2. Study Design

This manuscript was developed as a narrative, mechanism-oriented review aimed at integrating experimental biomechanical findings with clinically relevant considerations in fifth metatarsal fracture fixation. The objective was not to conduct a systematic or exhaustive review of all published studies, but rather to synthesize conceptually relevant literature that clarifies the mechanical principles underlying fixation behavior. Literature selection was guided by thematic relevance, focusing on studies addressing gait-phase loading, interfragmentary shear, cyclic fatigue, construct mechanics, and clinical fixation outcomes. Emphasis was placed on experimental investigations that provide mechanistic insight into bending, torsional, and shear-related failure modes, as well as clinical studies contextualizing these biomechanical observations.

Two of the biomechanical studies discussed in detail [16,17] were conducted by the present research group and are included as illustrative examples of methodological capability and full-field strain analysis techniques. These studies are presented within the broader context of existing literature and are not intended to represent a comprehensive or exclusive evidentiary basis. Accordingly, the findings and interpretations presented herein should be understood as hypothesis-generating and concept-integrative rather than prescriptive or guideline-forming.

## 3. Biomechanical Mechanisms Underlying Clinical Failure in Stress Fractures

### 3.1. High Bending Stresses

During gait, especially in the midstance phase, forces that are encountered in Zone II–III produce high bending. The fifth metatarsal, which serves as lateral support of the forefoot, endures forces that increase with foot misalignment. Studies analyzing biomechanics of sporting movements, as well as plantar loading patterns, have provided evidence that even small changes in the location of the foot can result in significant increases in shear forces applied to the fifth metatarsal; specifically, those patients with a cavus foot type or varus hindfoot alignment [16,17,18,19]. Because bending stresses are concentrated at the metaphyseal–diaphyseal cortex, this region becomes the “weak point” where microfractures typically occur. Over time, these injuries induced by jumping, pivoting, or sprinting motions generate torsional forces along the lateral column, producing the Zone II–III stress fracture pattern. Torsion is especially problematic because it has a combined effect with bending: bending concentrates stress on one cortex while torsion spreads stress around the bone. This combination amplifies the rate of microfracture appearance. In sports with quick starts and stops, athletes often use the forefoot to change direction, repeatedly twisting the fifth metatarsal. Consequently, the fifth metatarsal experiences repeated twisting loads.

### 3.2. Hypovascularity of Zone II–III

The metaphyseal–diaphyseal junction of the fifth metatarsal is known anatomically as a poor vascular area. With limited blood supply, osteoblastic activity is slower, and repair of microdamage is delayed compared to other foot bones. When repetitive stress is applied to a region with limited healing capacity, microfracture propagates faster. This contributes directly to delayed union, non-union, and refracture, especially in athletes who resume high-impact activities prematurely. Hypovascularity, therefore, transforms what might otherwise be a manageable fatigue injury into a chronic, structurally compromised condition [14,20].

### 3.3. Subtle Radiographic Onset

Stress fractures at the beginning stage, when only microfractures are formed, only MRI or CT can detect them. Early in their evolution, these fractures may not be visible on plain radiographs because cortical disruption has not yet occurred. MRI remains the gold standard for early detection, identifying bone marrow edema and subtle stress reactions before a visible fracture line develops. CT, on the other hand, is useful for assessing cortical thickening, incomplete fracture lines, or medullary canal widening in more advanced cases [21,22,23].

Advanced imaging provides clinically relevant proxies for biomechanical risk. Computed tomography can identify medullary canal widening, cortical thinning, and sclerosis, features associated with reduced screw purchase and increased stress concentration at the bone–implant interface. Magnetic resonance imaging identifies bone marrow edema and stress reactions, reflecting compromised bone subjected to repetitive loading. These radiologic findings may indicate a mechanically vulnerable environment in which compression-based fixation is more likely to fail under cyclic shear.

### 3.4. Clinical Implication

Because stress fractures result from cyclic loading rather than a single acute force, fixation implants should tolerate repetitive shear and bending, not just static loads. Traditional implants designed for compressive stabilization are insufficient if they cannot withstand the dynamic environment that produced the fracture in the first place. Implants should be evaluated not only for initial stiffness but also for fatigue resistance, torsional stability, and their ability to maintain alignment despite micro-gaps or cortical irregularities. This requirement is consistent with findings from recent 3D-printed biomechanical investigations, which will be further presented. They demonstrated that multidirectional loading, gap size, and loading angle (α) strongly influence stress distribution and implant performance [24,25]. These findings reinforce the need for fixation methods that can endure cyclical shear-dominant forces when treating stress fractures in high-demand patients.

### 3.5. Clinical Modifiers of Biomechanical Failure

Although biomechanical loading patterns are consistent, the clinical risk of fixation failure varies substantially between patients. Several patient- and fracture-specific factors modify the mechanical environment of the fifth metatarsal and influence construct performance.

*Foot alignment plays* a critical role. Cavovarus alignment and hindfoot varus increase lateral column loading and bending moments during midstance, amplifying shear forces acting on Zone II–III fractures.

*Patient activity level* is equally relevant. Athletes and physically active individuals expose fixation constructs to a higher number of loading cycles per day, accelerating fatigue-related failure mechanisms.

*Bone morphology*, particularly medullary canal widening or cortical thinning observed in stress fractures, reduces screw purchase and increases sensitivity to interfragmentary gaps.

*Timing of return to weight-bearing* further modulates mechanical risk. Early exposure to midstance-dominant loading may exceed the fatigue tolerance of compression-dependent constructs before biological healing occurs.

## 4. 3D Printing and DIC: A New Standard for Metatarsal Biomechanics

DIC measures surface strain and displacement by providing a whole-field view of the surface strain distribution over the bone-implant construct through an optical method without contact, in contrast to point-based measurement techniques like strain gauges, which measure the stress at one location only and therefore are limited to use in applications where the stress distribution is homogeneous or simple. Thus, DIC is ideal for applications involving complex geometries such as the fifth metatarsal. When DIC is used to study three-dimensional printed anatomically correct models of bones, it can provide a high degree of accuracy in identifying strain concentration at locations critical to the function of the model, such as at the interfaces between screws and bone, at the edges of plates, at the locations where hooks anchor into the bone, and at fracture gaps [23,26].

More recently, researchers have developed a new experimental design for the study of bone repair using DIC in conjunction with gait-phase-dependent loading protocols to create experimentally based conditions that mimic physiological conditions. The combination of DIC and gait-phase-dependent loading protocols has allowed researchers to evaluate how fixation constructs perform under multidirectional cyclic stresses during different phases of the stance cycle of gait. Specifically, this includes the phase of the stance cycle known as midstance, when lateral bending and shearing stresses reach their maximum levels and act upon the fifth metatarsal. Additionally, researchers have used factorial designs to vary the angle of loading (α), the magnitude of loading and the size of the interfragmentary gap. This has allowed researchers to assess how fixation constructs respond to various combinations of multidirectional cyclic stresses [16,24].

This integrated approach provides biomechanical insight into fatigue behavior, failure mechanisms, and construct stability under realistic conditions, bridging the gap between simplified laboratory testing and in vivo loading environments.

The experimental body of literature evaluating the use of PolyJet-based 3D printing and full-field strain measurements for fifth metatarsal fractures is very limited at the current time. The authors are aware of a few studies that have evaluated this methodology in detail [23,27].

The two experimental studies discussed herein, conducted by the present research group, are therefore included as illustrative case studies to demonstrate the capabilities, limitations, and potential translational value of such models, rather than as a comprehensive representation of the entire field. Two recent 3D-printed biomechanical investigations specifically evaluated fixation strategies for fifth metatarsal fractures under multidirectional loading conditions. The inclusion of the authors’ own biomechanical studies is intended to illustrate methodological capabilities and mechanistic insights rather than to imply construct superiority or clinical preference [16,17]. Both studies demonstrated that loading angle, interfragmentary gap size, and fixation construct strongly influence stress distribution and failure patterns. These models are constructed using CT-derived anatomical data to ensure accurate reproduction of cortical thickness, curvature, and metaphyseal–diaphyseal transitions. A major advantage of 3D-printed models is the replicability and the control of fracture. This is rarely achievable in cadaveric bone because of natural variability in morphology and tissue quality.

**Advantages for stress fracture modeling:**

- *Simulation of progressive microfracture propagation.* Controlled fracture lines can replicate early stress reactions, longitudinal cracks, or cortical breakthroughs, allowing detailed assessment of implant behavior in compromised bone.
- *High repeatability for fatigue research*. Fatigue studies require identical specimens subjected to identical loads that cannot be achieved with cadavers.
- *DIC detection of microstrain concentrations.* DIC provides high-resolution data, identifying localized stress peaks at screw threads, plate edges, and fracture gaps, which are common sites of clinical failure.

**Figure 3:**
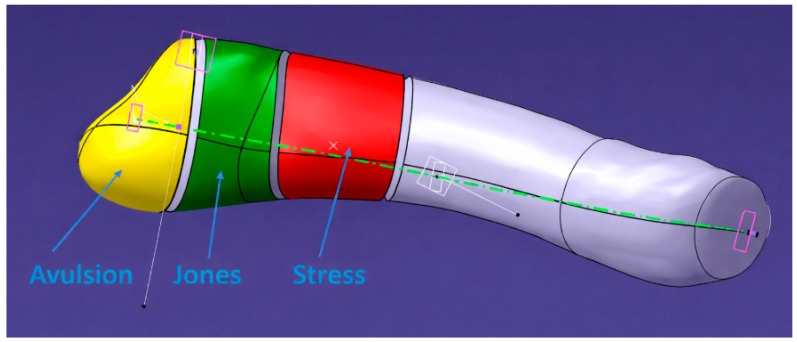
The figure illustrates different types of fractures of the fifth metatarsal, highlighted using color coding: yellow—Avulsion fracture: located at the proximal tuberosity, caused by traction from a tendon or ligament. Green—Jones fracture: situated at the metaphyseal–diaphyseal junction, distal to the avulsion zone; red—Stress fracture: located in the proximal diaphyseal region, typically caused by repetitive mechanical loading; **green dashed line:** represents the longitudinal axis of the bone; letter “X” (at the distal right end): indicates the reference point from which the geometric modeling of the bone begins in the 3D representation.

**Figure 4:**
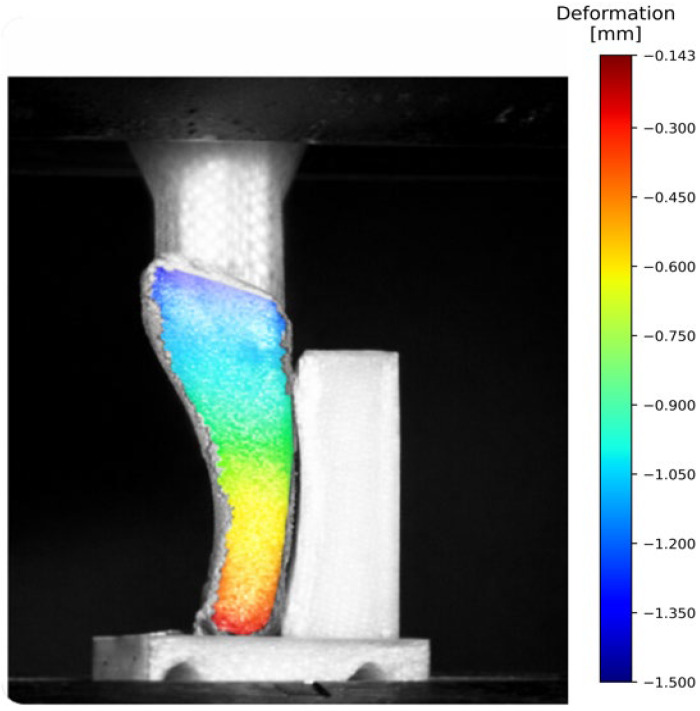
Full-field displacement map obtained by DIC on a 3D-printed fifth metatarsal fracture model under simulated gait-phase loading.

The experimental procedure used within the studies described above is outlined schematically in Figure 3, while a sample of full-field deformation maps produced by DIC analysis is presented in Figure 4.

Beyond these advantages of the combined use of 3D printing and DIC, researchers can now recreate physiologically relevant, multi-axial loading conditions that mirror gait. Testing using different loading angles (α), inversion forces or plantarflexion–dorsiflexion orientation can demonstrate which surgical techniques are viable in different situations.

The experimental studies utilizing 3D printed PolyJet models with high-resolution DIC have provided significant mechanical data. Nevertheless, it is also necessary to acknowledge the limitations of the methodology used. The models utilized in the studies do not accurately represent the anisotropic and viscoelastic characteristics of human bone. In addition, there was no representation of biological healing processes, vascular responses or soft tissue constraints on the models utilized. Therefore, they cannot be directly applied to clinical settings. Due to the high repeatability of the models, this increases the internal validity of the study; however, the external validity remains limited. As a result, data generated using these methodologies should be considered as providing a mechanistic explanation for results rather than predicting clinical outcomes.

Data from other anatomical sites has indicated that 3D-based surgical planning and patient-specific modeling can enhance the accuracy and safety of surgical interventions, especially those involving complex spinal and coxofemoral anatomy. Data suggest the general applicability of precision modeling approaches and motivate further validation of similar workflow applications in smaller load-bearing bones [17,25].

## 5. Clinically Relevant Biomechanical Lessons from Avulsion and Jones Fracture Fixation

It is essential to recognize that two of the biomechanics studies presented in detail were conducted by the current authors. They are presented in this report not as evidence of a clinical advantage, but as examples of experimental frameworks that demonstrate the value of controlled and repeatable biomechanical testing. Wherever possible, the conclusions drawn from the studies are compared to data generated from independent biomechanics and clinical literature to limit potential biases in interpretation.

### 5.1. Multidirectional Loading Is the Key Determinant of Construct Stability

Both studies demonstrated that the loading angle (α) is the most important factor that influences the yielding of osteosynthetic material. This finding is clinically important because foot loading during gait is highly dynamic: forces shift from dorsiflexion to plantarflexion across stance phases.

During midstance, the fifth metatarsal experiences maximal lateral bending and inversion stresses, precisely the conditions under which stress fractures develop and propagate. This explains why certain implants that appear adequate perform poorly under physiologic cyclic loading: they were never designed to tolerate repeated shear peaks that occur during midstance.

The strong influence of loading angle also highlights an important point for surgeons: fixation constructs must be evaluated not only based on their static stiffness but also in terms of how they redistribute loads throughout the gait cycle. Understanding these aspects can determine the moment when the implant fails or the risk of refracture is very high.

### 5.2. Plate-Based Constructs Demonstrate Improved Mechanical Behavior Under Shear and Imperfect Reduction Conditions

Findings from both studies show:

- Hook plates reduce peak stresses in avulsion fracture models.
- T-plates significantly increase failure thresholds in Jones fracture models.
- Intramedullary or bicortical screws are highly sensitive to interfragmentary gap and show early stress concentration.

These results reflect that screws rely on compression to stabilize fracture fragments. Similar biomechanical observations have been reported in independent experimental studies. Bean et al. demonstrated improved resistance to multidirectional loading with hook-plate constructs compared to compression screws in large avulsion fractures, particularly under rotational stress. Likewise, clinical series in high-demand athletic populations have reported favorable outcomes with plate fixation in cases of revision surgery or cortical compromise [26,27,28,29].

When a gap between fragments after fixation is present or when cortical thinning reduces thread engagement, the interface between screw and bone experiences high localized tensions. Under repeated shear loading, these stress peaks accelerate microdamage and can lead to failure of the implant.

In contrast, plates distribute load and provide superior bending rigidity. Rather than concentrating forces at a single thread interface, plates disperse stresses. This behavior is advantageous for stress fractures, which often present with:

- Cortical widening,
- Longitudinal fracture propagation,
- Incomplete fracture lines.

Because perfect reduction is rarely achievable surgically and because bone quality is often compromised by repetitive loading, plate-based constructs may provide mechanical advantages in scenarios characterized by shear-dominant loading, cortical compromise, or imperfect reduction. They tolerate malalignment better, maintain stiffness despite small gaps between fragments and resist multidirectional shear more effectively [27,28,29].

To facilitate comparison across fixation strategies, Table 1 summarizes the key biomechanical characteristics reported in the experimental and clinical literature.

While the experimental data presented herein provide mechanistic insight, similar principles regarding shear sensitivity and gap dependence have been discussed in broader orthopedic biomechanics literature, particularly in studies evaluating compression-dependent constructs under cyclic loading. These findings support the generalizability of the mechanical concepts described, beyond the specific models utilized in this review.

**Table 1:** Comparative biomechanical characteristics of fixation constructs.

Construct	Bending Resistance	Shear Tolerance	Gap Sensitivity	Fatigue Resistance	Clinical Implication
Intramedullary screw [16,28]	Moderate	Low	High	Limited	Early fractures, intact canal
Bicortical screw [27,29]	Moderate	Low–moderate	High	Limited	Requires perfect reduction
Plate fixation [16,24,27,29]	High	High	Low	High	Stress fractures, athletes
Hybrid constructs [15,16,17]	Very high	High	Low	Very high	Refracture, nonunion

These qualitative categorizations are intended to summarize trends observed across heterogeneous experimental and clinical studies and do not represent standardized quantitative mechanical thresholds. These biomechanical findings help explain the higher rates of delayed union and refracture reported with screw fixation in patients with cortical widening, imperfect reduction, or high cyclic loading demands. In such scenarios, plate-based constructs may offer greater mechanical robustness under experimental conditions.

### 5.3. Nonlinear Interactions Between Angle and Gap Explain Clinical Failure Patterns

The interaction between loading angle and distance between fragments offers a clear explanation for clinical screw failures. These failures often occur not because the implant is weak, but because small distances between fragments (even 0.1 mm gaps) can amplify bending and shear forces. However, because restoring full cortical continuity is often challenging, plate fixation or hybrid constructs combining screw compression with plate provide more long-term stability.

### 5.4. Potential Role of Hook Plates in Stress Fracture Fixation

Although hook plates are traditionally used for Zone I fractures, *biomechanically, they are relevant* for zones II–III because:

- Hook plates provided the most uniform stress dispersion.
- They controlled rotational and shear forces better than screws.
- Their multi-point fixation could stabilize the metaphyseal cortex.

The uniform stress distribution achieved with hook plates suggests they can have applicability in zone III fractures because athletes can have microfractures along the lateral cortex, so the hook plate can provide them with better stabilization.

Although hook plates are not routinely used for stress fractures, the mechanical behavior demonstrated in both studies suggests that they can provide a better fixation in some cases, when other stress reactions coexist, when tuberosity is involved or when cortical stability is compromised.

It should also be noted that the experimental data included in this section derive from an extremely limited number of studies. These papers were never intended to provide a complete review of the literature but instead were to be used as examples of the mechanics of implant fixation under controlled conditions. Limitations of the studies mentioned above arise from their experimental design and, therefore, should not be viewed as proof that one method of fixation will result in better outcomes for patients than other methods in a clinical setting.

### 5.5. Illustrative Clinical Scenarios

*Scenario 1:* A high-level athlete with a Jones fracture and subtle medullary canal widening on CT may achieve initial stability with intramedullary screw fixation; however, biomechanical data suggest increased sensitivity to shear during early return to play, favoring plate-based constructs.

*Scenario 2:* A recreational athlete with an acute Jones fracture, intact cortical morphology, and delayed weight-bearing may benefit from screw fixation, provided that anatomic reduction and sufficient compression are achieved.

*Scenario 3:* A patient with recurrent stress fracture and cortical sclerosis represents a mechanically compromised environment in which plate or hybrid fixation may provide superior fatigue resistance.

## 6. Future Implants for Stress Fracture Using 3D-Based Evidence

Stress fractures occur in a high-bending environment. The fifth metatarsal experiences substantial lateral bending moments during midstance. Any fixation construct must therefore have the mechanical properties that can avoid tension concentrations.

Ideally, implants should have the following characteristics:

- Implants that offer rigidity to the lateral column, ensuring that constructs resist without excessively stiffening the bone–implant interface. A balanced stiffness profile would allow physiologic load transfer while preventing excessive tension at the fracture site.
- Hybrid constructs combining screw compression with lateral plate have the advantages of axial compression (screw) with the superior bending resistance of plates. Such constructs could theoretically provide optimal stability throughout the gait cycle.
- Patient-specific plate obtained by 3D printing. The implants are adapted to the unique curvature and cortical thickness of each individual, reducing implant prominence, improving load sharing and potentially decreasing irritation of surrounding soft tissues.

Future implant systems for fifth metatarsal stress fractures will likely integrate these concepts, providing a new generation of fixation devices. Combining personalized anatomy with fatigue-resistant mechanics, such designs hold potential to minimize refracture risk and optimize outcomes for athletes and active individuals [24,25].

## 7. Clinical Implications Across All Three Fracture Types

The biomechanical principles discussed above have direct clinical relevance across all three major fifth metatarsal fracture patterns and may assist surgeons in tailoring fixation strategies to patient-specific mechanical and biological conditions. Recent 3D-printed biomechanical studies using PolyJet models combined with digital image correlation (DIC) have enabled full-field strain quantification under simulated gait-phase loading, improving understanding of how multidirectional cyclic stress, imperfect reduction, and fatigue influence construct behavior.

More recently, 3D-printed biomechanical studies utilizing PolyJet models combined with DIC have allowed researchers to measure full-field strains in the implant–bone construct under simulated gait-phase loading conditions [16,24]. The biomechanical principles demonstrated in these studies support clinically meaningful interpretation for avulsion fractures, Jones fractures, and stress fractures of the fifth metatarsal by clarifying construct sensitivity to loading direction, interfragmentary gap, and cyclic fatigue [30,31]. However, biomechanical advantages observed in controlled experimental conditions should not be interpreted as definitive evidence of clinical superiority without comparative prospective validation.

**Avulsion fractures (Zone I).** Hook plates may provide mechanical advantages in displaced or unstable avulsion fractures by distributing loads across a broader surface and reducing stress concentration at the fragment–implant interface under multidirectional loading. This mechanism is consistent with their observed clinical performance in cases where tendon pull contributes to displacement and early functional demands are high [32].

**Jones fractures (Zone II).** Experimental evidence indicates that plate constructs may demonstrate improved resistance to cyclic shear and bending compared with compression-dependent screw fixation, particularly when reduction is imperfect or a small interfragmentary gap persists. This is clinically relevant in the hypovascular Zone II environment, where biological healing potential may be limited and fixation stability becomes critical [19,33,34,35]. Accordingly, plate-based constructs may be considered in high-demand patients, in displaced fractures, or when reduction quality and gap closure are uncertain, while acknowledging that screw fixation remains widely used and often successful in appropriately selected cases.

**Stress fractures (Zone II–III).** Stress fractures originate from repetitive microtrauma; therefore, construct selection should account for fatigue behavior rather than only initial stiffness. The 3D-printed cyclic loading models consistently demonstrate that plate-based constructs may maintain stability under cyclic shear and tolerate cortical irregularities and micro-gaps more effectively than compression-dependent strategies in certain experimental conditions [32,33]. Importantly, intramedullary screw fixation remains an effective and clinically accepted option in selected athletic populations—particularly in early-stage injuries with preserved endosteal integrity, minimal canal widening, and appropriate postoperative management—with multiple clinical series reporting high union rates and rapid return to sport under such conditions [19,28,33]. When medullary canal widening, cortical thinning, or longitudinal crack patterns compromise screw purchase and increase shear sensitivity, plate-based constructs may represent mechanically favorable options in that context [32,33].

Importantly, biomechanical construct behavior represents only one dimension of clinical outcome, which is additionally influenced by biological healing capacity, rehabilitation protocols, and patient-specific variability. Furthermore, the number of independent full-field strain investigations specifically addressing fifth metatarsal fracture fixation remains limited. As a result, mechanistic interpretations currently rely on a relatively small body of experimental work, underscoring the need for broader external validation.

## 8. Integrating Biomechanics into Surgical Decision-Making

The following points are intended as biomechanics-informed considerations to support clinical judgment and individualized planning, rather than algorithmic rules for practice. Because the mechanical environment of the lateral foot column varies across fracture types and stages of injury, fixation strategies should be selected with attention to both biological context and expected gait-related loading.

Evidence from clinical practice and 3D-printed full-field strain analyses suggests that construct performance is influenced by fracture morphology, reduction quality, and the dominant loading mechanism during gait [16,24]. Several factors should be considered when selecting an appropriate fixation strategy:

- **Fracture type** (avulsion vs. Jones vs. stress fracture), which determines the prevailing failure mechanism and biological environment.
- **Dominant loading mechanism**, as constructs that perform well under compression may show vulnerability under cyclic shear, particularly during midstance.
- **Quality of achievable reduction**, because even small interfragmentary gaps may increase strain concentration at the interface under cyclic loading.
- **Athletic demand and return-to-activity timing**, which increase fatigue cycling and may amplify shear-related failure risk.
- **Cortical and canal morphology** (e.g., widening, thinning, sclerosis), which can reduce screw purchase and increase sensitivity to loading direction.

These considerations support a conceptual decision-support framework grounded in both biological and mechanical principles, rather than a prescriptive treatment algorithm. In stress fractures without canal widening and with preserved endosteal integrity, screw-based fixation (with or without adjuncts such as grafting) may be appropriate; in stress fractures with cortical compromise or widening, plate-based constructs may provide mechanical advantages under cyclic shear and bending conditions in experimental models [16,24]. In refracture or chronic nonunion scenarios, hybrid strategies may be considered to improve load sharing and fatigue resistance, while acknowledging that high-quality comparative trials remain limited. Due to the lack of well-designed prospective comparative clinical trials directly evaluating fixation constructs under standardized conditions, biomechanical findings should be interpreted as mechanistic explanations that complement—rather than replace—clinical outcome evidence and surgeon experience. This conceptual biomechanics-informed framework is intended to support individualized decision-making and reduce fixation failure risk across fifth metatarsal fracture patterns [36]. These interpretations should be considered conceptual extrapolations from controlled experimental settings rather than definitive comparative clinical evidence. It must be emphasized that these interpretations derive primarily from controlled experimental models and conceptual integration of heterogeneous literature. They should therefore be regarded as biomechanical rationale rather than evidence of comparative clinical superiority.

## 9. Limitations of Biomechanical and Clinical Evidence

We acknowledge that a proportion of the experimental research that we have reviewed in this manuscript was performed by us. Therefore, there exists the possibility of selection and interpretation biases towards certain experimental designs and fixation constructs. Therefore, we consider the results of our own experimental work as case study examples of a mechanistically driven explanation for the benefits of certain fixation methods and not as definitive evidence of clinical superiority. We attempt to place our results within the larger context of the independent biomechanical and clinical literature available to reduce the likelihood of overemphasizing the results of our own studies.

Biomechanical models, such as 3D-printed bone surrogates, assume that the materials used to simulate bone exhibit uniform properties (i.e., homogeneous) and isotropic properties, and also simplify boundary conditions (the effects of surrounding soft tissues). Although these models allow precise manipulation of fracture geometry and direction of load application, they do not replicate the biological heterogeneity of bone, nor the process of bone remodeling after injury, nor the contributions of soft tissues to the overall structural behavior of the bone.

The PolyJet material used in the experimental models exhibits elastic modulus values significantly lower than human cortical bone (reported range for cortical bone: ~7–20 GPa) and lacks anisotropic and viscoelastic characteristics. Therefore, the models serve primarily mechanistic and comparative purposes rather than absolute load prediction. Consequently, absolute stress magnitudes, fatigue thresholds, and construct endurance observed in these models cannot be directly extrapolated to in vivo conditions but rather serve comparative and mechanistic purposes.

In addition, clinical studies of fifth metatarsal fractures are limited by the fact that patients who undergo surgical treatment often represent a diverse population with respect to demographic characteristics, type and extent of injury, preoperative activity level, postoperative rehabilitation protocol, etc. Moreover, many clinical reports use varying definitions of “union” and “failure”, and the majority of studies are retrospective in nature and too small in size to detect statistically significant differences between various fixation constructs.

As such, the results of biomechanical studies should be viewed as explanatory, rather than predictive, and used as complementary information to clinical outcomes data in guiding clinical decisions.

Although the existing body of evidence regarding fixation strategies for fifth metatarsal fractures includes both biomechanical and clinical studies, the body of evidence is still heterogeneous. In particular, most biomechanical studies, including those using 3D-printed models, make several simplifying assumptions about the bone and its behavior under load, and conduct experiments under tightly controlled laboratory conditions that cannot possibly reflect the full range of variability that can occur in patients, including variability due to bone remodeling, rehabilitation practices, etc.

Furthermore, although most clinical studies of fifth metatarsal fractures are retrospective in design, include relatively small numbers of subjects, and use non-uniform criteria for defining successful vs. unsuccessful treatment (whether the patient achieved “union,” failed to achieve “union,” etc.), clinical studies are the primary source of clinical evidence in this area, and therefore caution is warranted when attempting to translate the results of biomechanical studies into clinical practice. Finally, while the zonal classification of fifth metatarsal fractures illustrated in Figure 1 provides a biomechanically oriented framework for understanding the mechanical stresses experienced at different locations along the length of the fracture, this classification scheme does not account for the variability in bone geometry, cortical thickness, or foot alignment that can affect the magnitude and distribution of mechanical stresses at the fracture site.

To date, no randomized controlled trials directly compare intramedullary screw, plate, and hybrid constructs specifically in the context of stress-related fifth metatarsal fractures under standardized conditions. Additionally, long-term implant survivorship data and fatigue-related outcome studies remain limited. The relatively limited number of independent full-field strain investigations in fifth metatarsal models constrains the breadth of currently available mechanistic validation.

## 10. Clinical Decision-Making Implications

From a clinical standpoint, fixation failure in fifth metatarsal fractures is rarely the result of a single factor, but rather the interaction between fracture morphology, patient-specific loading demands, and the mechanical behavior of the chosen fixation construct. The biomechanical evidence summarized in this review provides a framework that may assist surgeons in tailoring fixation strategies to individual clinical scenarios rather than relying on a one-size-fits-all approach.

Patient-related factors play a critical role in construct performance. High-demand patients, such as athletes or individuals who return early to weight-bearing activities, expose fixation constructs to repetitive cyclic loading and multidirectional forces during gait. In these settings, constructs that rely predominantly on interfragmentary compression may be more susceptible to loss of stability under shear and bending forces, particularly during early rehabilitation.

Fracture-related characteristics further influence mechanical risk. Jones fractures and diaphyseal stress fractures combine hypovascularity with repetitive midstance loading, predisposing them to delayed union and refracture. Features such as cortical widening, sclerosis, or small residual fracture gaps—commonly encountered in stress-related injuries—may reduce the effectiveness of compression-based fixation and increase sensitivity to loading direction.

Fixation strategy considerations should therefore account for both mechanical and biological factors. Intramedullary screw fixation may be appropriate in acute fractures with good bone quality and minimal gap formation, particularly in lower-demand patients. In contrast, plate-based or hybrid constructs may offer greater resistance to cyclic shear and bending, improved tolerance to imperfect reduction, and more uniform load distribution across the fracture site. These characteristics may be advantageous in high-demand patients, stress fractures, revision cases, or situations in which early mechanical stability is prioritized.

Rather than advocating a single optimal fixation method, the available biomechanical and clinical evidence supports an individualized, biomechanics-informed approach to surgical decision-making. Integrating patient activity level, fracture morphology, and expected loading conditions may help reduce fixation failure and refracture risk in the treatment of fifth metatarsal fractures.

Although primarily biomechanical in focus, fixation outcomes are also influenced by broader biological and neurophysiological factors, which justify brief consideration. Beyond structural and mechanical determinants, pain sensitization and neuromuscular adaptations may influence postoperative loading patterns and rehabilitation tolerance. Central sensitization mechanisms can alter weight-bearing behavior, gait symmetry, and muscular activation patterns, potentially modifying the distribution and magnitude of forces transmitted across the fixation construct. Although these neurophysiological factors are not specific to fifth metatarsal fractures, they may indirectly interact with mechanical stability and healing by influencing functional loading conditions during recovery. Therefore, fixation outcomes should be interpreted within a broader biopsychosocial context, particularly in high-demand or chronic cases [37].

Emerging evidence suggests a complex interaction between physical activity, systemic inflammatory responses, and bone metabolism, potentially mediated through gut microbiota. Although these systemic biological factors were beyond the primary biomechanical scope of the present review, they may influence bone remodeling capacity, inflammatory modulation, and fracture healing efficiency, thereby indirectly interacting with the mechanical environment in which fixation constructs operate [38].

Intramedullary screw fixation offers important clinical advantages beyond biomechanics, including minimally invasive insertion, preservation of soft tissue envelope, shorter operative time, and simplified revision strategies. These factors contribute to its continued widespread use and favorable outcomes in selected patient populations.

These considerations are intended to complement clinical outcome data and surgeon experience rather than replace them.

## 11. Conclusions

This narrative, mechanism-oriented review integrates experimental biomechanics with clinically relevant considerations in fifth metatarsal fracture fixation. Available evidence suggests that construct performance may vary depending on fracture morphology, reduction quality, and loading environment, particularly under cyclic shear and bending conditions.

However, experimental models inherently simplify biological complexity and cannot fully replicate patient-specific variability, bone remodeling processes, or rehabilitation influences. Current clinical evidence remains heterogeneous and largely retrospective, with limited prospective comparative data directly evaluating fixation constructs under standardized conditions.

Accordingly, the biomechanical insights presented should be interpreted as hypothesis-generating and complementary to clinical judgment rather than prescriptive guidance. Individualized fixation strategies that account for fracture characteristics, mechanical loading demands, and patient-specific factors remain essential. Well-designed prospective comparative studies are required to determine whether experimentally observed mechanical differences translate into meaningful improvements in clinical outcomes.

## Data Availability

The data supporting the findings of this study are available from the corresponding author upon reasonable request.
